# Description of a new horned toad of *Megophrys* Kuhl & Van Hasselt, 1822 (Amphibia, Megophryidae) from Zhejiang Province, China

**DOI:** 10.3897/zookeys.1005.58629

**Published:** 2020-12-18

**Authors:** Yanqing Wu, Shize Li, Wei Liu, Bin Wang, Jun Wu

**Affiliations:** 1 Nanjing Institute of Environmental Sciences, Ministry of Ecology and Environment of China, Nanjing 210042, China Nanjing Institute of Environmental Sciences, Ministry of Ecology and Environment of China Nanjing China; 2 Chengdu Institute of Biology, Chinese Academy of Sciences, Chengdu 610041, China Chengdu Institute of Biology, Chinese Academy of Sciences Chengdu China; 3 Lishui Baiyun Ecological Forest Farm, Lishui 323000, China Lishui Baiyun Ecological Forest Farm Lishui China

**Keywords:** Molecular phylogenetic analyses, morphology, new species, taxonomy, toad

## Abstract

A new species of the Asian horned toad genus *Megophrys* is described from Zhejiang Province, China, based on multiple data. Molecular phylogenetic analyses based on mitochondrial DNA indicated the new species as an independent clade deeply clustered into the *Megophrys* clade. The new species is identified from its congeners by a combination of the following characters: body size small (SVL 28.4–32.4 mm in males); vomerine teeth absent; tongue not notched behind; tympanum distinctly visible, oval; a small horn-like tubercle present at the edge of each upper eyelid; two metacarpal tubercles distinctly visible in hand; toes without webbing; heels overlapped when thighs are positioned at right angles to the body; tibiotarsal articulation reaching the level to middle of eye when leg stretched forward; an internal single subgular vocal sac in male; in breeding male, the nuptial pads present on the dorsal base of the first two fingers.

## Introduction

The Asian horned toad *Megophrys* Kuhl & Van Hasselt, 1822 (Anura: Megophryidae Bonaparte, 1850) is widely distributed in eastern and central China, throughout southeastern Asia, and extending to the islands of the Sunda Shelf and the Philippines (Frost 2020). The generic assignment of species in the group has been controversial for decades (e.g., Tian and Hu 1983; Dubois 1987; Rao and Yang 1997; Lathrop 1997; Jiang et al. 2003; Delorme et al. 2006; Fei et al. 2009; Fei and Ye 2016; Chen et al. 2017; Deuti et al. 2017; Mahony et al. 2017; Li et al. 2020). Recent molecular phylogenetic studies proposed this group as a monophyletic group (Chen et al. 2017; Mahony et al. 2017; Li et al. 2018a; Liu et al. 2018; Liu et al. 2020; Wang et al. 2020), which was recognized as a big genus *Megophryssensu lato* (Mahony et al. 2017; Li et al. 2018b; Liu et al. 2018; Liu et al. 2018; Liu et al. 2020; Lyu et al. 2020; Xu et al. 2020; Wang et al. 2020), though some studies still divided the taxa of the group into different genera and/or subgenera (Fei and Ye 2016; Chen et al. 2017; Deuti et al. 2017; Liu et al. 2018). The genus *Megophrys* currently contains 106 species, of which 52 species were described over the last decade (Frost 2020). A number of cryptic species were still indicated in the genus by molecular phylogenetic analyses (e.g., Chen et al. 2017; Liu et al. 2018).

Wuyi Mountain region, located in northern Fujian, southeastern Jiangxi and south Zhejiang provinces of China, is a biodiversity hotspot. In this region, four *Megophrys* species have been recorded, i.e., *M. boettgeri* (Boulenger, 1899), *M. kuatunensis* Pope, 1929, *M. ombrophila* Messenger & Dahn, 2019, and *M. lishuiensis* Wang, Liu & Jiang, 2017. However, many mountains in this region, especially in south Zhejiang Province, have been poorly investigated.

During field surveys in Qingyuan County, Zhejiang Province, China, we collected *Megophrys* specimens. Molecular phylogenetic analyses and morphological comparisons supported some of these specimens as an undescribed taxon that we describe herein as a new species.

## Materials and methods

### Sampling

**Figure 1.**
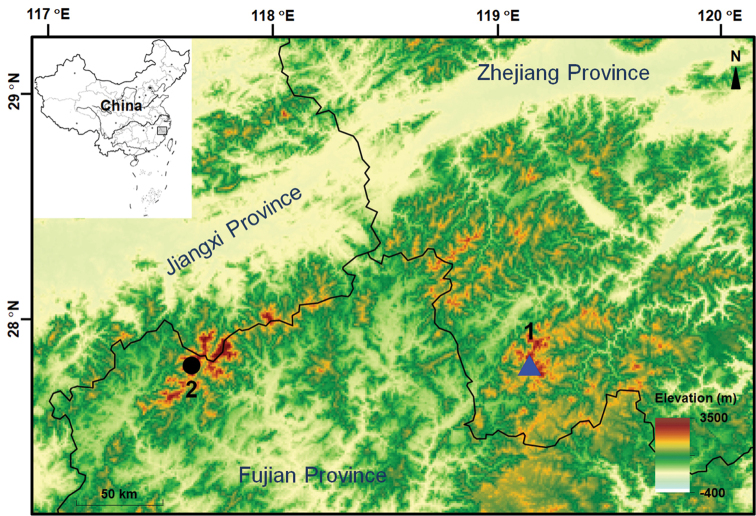
Sampling localities of *Megophrys baishanzuensis* sp. nov. and its relatives **1** Baishanzu National Park, Qingyuan County, Zhejiang Province, China, inhabited by *Megophrys baishanzuensis* sp. nov. and *M. boettgeri***2** Wuyi Mountain, Wuyishan City, Fujian Province, China, inhabited by *M. boettgeri*, *M. kuatunensis*, and *M. ombrophila*.

A total of 15 specimens were sampled in this study: six adult males and one tadpole of the undescribed species and two adult males of *M. boettgeri* from Qingyuan County, Zhejiang Province, China, and one adult male of *M. ombrophila* and six adult males of *M. kuatunensis* from Wuyi Mountain, Fujian Province, China (Table 1; Fig. 1). The developmental stage of tadpole was identified following Gosner (1960). In the field, the toad and tadpole were euthanized using isoflurane, and the specimens were fixed in 75% ethanol. Tissue samples were taken and preserved separately in 95% ethanol prior to fixation. The specimens were deposited in Chengdu Institute of Biology, Chinese Academy of Sciences (**CIB, CAS**).

**Table 1.** Information for samples used in molecular phylogenetic analyses in this study.

ID	Species	Voucher number	Locality	GenBank accession number	
				16S	COI
1	*Megophrys baishanzuensis* sp. nov.	CIBQY20200719001	Baishanzu National Park, Qingyuan, Zhejiang, China	MW001150	MT998291
2	*Megophrys baishanzuensis* sp. nov.	CIBQY20200719002		MW001151	MT998292
3	*Megophrys baishanzuensis* sp. nov.	CIBQY20200719003		MW001152	MT998293
4	*Megophrys baishanzuensis* sp. nov.	CIBQY20200719004		MW001153	MT998294
5	*Megophrys baishanzuensis* sp. nov.	CIBQY20200719006		MW001154	MT998295
6	*Megophrys baishanzuensis* sp. nov.	CIBQY20200726001		MW001155	MT998296
7	*Megophrys baishanzuensis* sp. nov.	CIBQY20200726002		MW001156	MT998297
8	*Megophrys kuatunensis*	CIBWY18082407	Wuyi Shan, Fujian, China	MW001157	MT998298
9	*Megophrys kuatunensis*	CIBWY18082408		MW001158	MT998299
10	*Megophrys kuatunensis*	SYS a001579		KJ560376	–
11	*Megophrys lini*	SYS a002370	Suichuan, Jiangxi, China	KJ560412	–
12	*Megophrys xiangnanensis*	SYS a002874	Yangming Shan, Hunan, China	MH406713	MH406165
13	*Megophrys nanlingensis*	SYS a001959	Nanling Nature Reserve, Guangdong, China	MK524111	MK524142
14	*Megophrys dongguanensis*	SYS a001972	Yinping Shan, Guangdong, China	MK524098	MK524129
15	*Megophrys nankunensis*	SYS a004498	Nankun Shan, Guangdong, China	MK524108	MK524139
16	*Megophrys cheni*	SYS a001427	Jinggang Shan, Jiangxi, China	KJ560391	–
17	*Megophrys wugongensis*	SYS a002610	Wugongshan Scenic Area, Jiangxi, China	MK524114	MK524145
18	*Megophrys ombrophila*	KRM18	Wuyishan, Fujian, China	KX856404	–
19	*Megophrys ombrophila*	CIBWY18082308		MW001159	MT998300
20	*Megophrys obesa*	SYS a002272	Heishiding Nature Reserve, Guangdong, China	KJ579122	–
21	*Megophrys lishuiensis*	WYF00169	Lishui, zhejiang, China	KY021418	–
22	*Megophrys xianjuensis*	CIBXJ190505	Xianju, zhejiang, China	MN563753	MN563769
23	*Megophrys jinggangensis*	KIZ07132	Chashan Forest Farm, Jiangxi, China	KX811840	KX812108
24	*Megophrys boettgeri*	CIB20200718001	Baishanzu National Park, Qingyuan, Zhejiang, China	MW001160	MT998301
25	*Megophrys boettgeri*	CIB20200718002	Baishanzu National Park, Qingyuan, Zhejiang, China	MW001161	MT998302
26	*Megophrys boettgeri*	Tissue ID: YPXJK033	Wuyi Shan, Fujian, China	KX811814	KX812104
27	*Megophrys huangshanensis*	KIZ022004	Huang Shan, Anhui, China	KX811821	KX812107
28	*Megophrys liboensis*	GNUG:20160408003	Libo, Guizhou, China	MF285262	–
29	*Megophrys mufumontana*	SYS a006391	Mufu Shan, Hunan, China	MK524105	MK524136
30	*Megophrys wushanensis*	KIZ045469	Guangwu Shan, Sichuan, China	KX811838	KX812094
31	*Megophrys baolongensis*	KIZ019216	Baolong, Chongqing, China	KX811813	KX812093
32	*Megophrys tuberogranulata*	Tissue ID: YPX10987	Badagongshan Nature Reserve, Hunan, China	KX811823	KX812095
33	*Megophrys yangmingensis*	SYS a002877	Yangming Shan, Hunan, China	MH406716	MH406168
34	*Megophrys shimentaina*	SYS a002077	Shimentai Nature Reserve Guangdong, China	MH406655	MH406092
35	*Megophrys jiulianensis*	SYS a002107	Jiulian Shan, Jiangxi, China	MK524099	MK524130
36	*Megophrys shunhuangensis*	HNNU16SH02	Shunhuang Mountains, Hunan, China	MK836037	–
37	*Megophrys mirabilis*	SYS a002192	Huaping Nature Reserve, Guangxi, China	MH406669	MH406109
38	*Megophrys leishanensis*	CIBLS20171101001	Leigong Shan, Guizhou, China	MK005310	MK005306
39	*Megophrys omeimontis*	KIZ025765	Emei Shan, Sichuan, China	KX811884	KX812136
40	*Megophrys angka*	KIZ040591	Kiew Mae Pan nature trail, Chiang Mai, Thailand	MN508052	–
41	*Megophrys binchuanensis*	KIZ019441	Jizu Shan, Yunnan, China	KX811849	KX812112
42	*Megophrys palpebralespinosa*	KIZ011603	Pu Hu Nature Reserve, Thanh Hoa, Vietnam	KX811888	KX812137
43	*Megophrys spinata*	SYSa002227	Leigong Shan, Guizhou, China	MH406676	MH406116
44	*Megophrys sangzhiensis*	SYSa004307	Zhangjiajie, Hunan, China	MH406798	MH406260
45	*Megophrys binlingensis*	SYSa005313	Wawu Shan, Sichuan, China	MH406892	MH406354
46	*Megophrys wuliangshanensis*	KIZ046812	Huangcaoling, Yunnan, China	KX811881	KX812129
47	*Megophrys daweimontis*	KIZ048997	Dawei Shan, Yunnan, China	KX811867	KX812125
48	*Megophrys jingdongensis*	KIZ-LC0805067	Huanglianshan National Nature Reserve, Yunnan, China	KX811872	KX812131
49	*Megophrys fansipanensis*	VNMN 2018.01	Lao Cai, Sa Pa, Vietnam	MH514886	–
50	*Megophrys hoanglienensis*	VNMN 2018.02	Lao Cai, Sa Pa, Vietnam	MH514889	–
51	*Megophrys minor*	KIZ01939	Qingcheng Shan, Sichuan, China	KX811896	KX812145
52	*Megophrys jiangi*	CIBKKS20180722006	Kuankuosui Nature Reserve, Guizhou, China	MN107743	MN107748
53	*Megophrys chishuiensis*	CIBCS20190518031	Chishui Nature Reserve, Guizhou, China	MN954707	MN928958
54	*Megophrys brachykolos*	ROM 16634	Hong Kong, China	KX811897	KX812150
55	*Megophrys acuta*	SYS a001957	Heishiding Nature Reserve, Guangdong, China	KJ579118	–
56	*Megophrys gerti*	ITBCZ 1108	Nui Chua National Park, Ninh Thuan, Vietnam	KX811917	KX812161
57	*Megophrys elfina*	ZMMU ABV-00454	Bidoup Mountain, Lam Dong, Vietnam	KY425379	–
58	*Megophrys synoria*	FMNH 262778	O’Reang, Mondolkiri, Cambodia	KY022198	–
59	*Megophrys hansi*	KIZ010360	Phong Dien Nature Reserve, Thua Thien Hue, Vietnam	KX811913	KX812155
60	*Megophrys microstoma*	KIZ048799	Xiaoqiaogou Nature Reserve, Yunnan, China	KX811914	KX812156
61	*Megophrys pachyproctus*	KIZ010978	Beibeng, Xizang, China	KX811908	KX812153
62	*Megophrys baluensis*	ZMH A13125	Gunung Kinabalu National Park, Kogopan Trail, Malaysia	KJ831310	–
63	*Megophrys stejnegeri*	KU 314303	Pasonanca Natural Park, Zamboanga, Philippines	KX811922	KX812052
64	*Megophrys ligayae*	ZMMU NAP-05015	Palawan, Philippines	KX811919	KX812051
65	*Megophrys kobayashii*	UNIMAS 8148	Gunung Kinabalu National Park, Sabah, Malaysia	KJ831313	–
66	*Megophrys nasuta*	KIZ019419	Malaysia	KX811921	KX812054
67	*Megophrys edwardinae*	FMNH 273694	Bintulu, Sarawak, Malaysia	KX811918	KX812050
68	*Megophrys aceras*	KIZ025467	Khao Nan National Park, Nakhon Si Thammarat, Thailand	KX811925	KX812159
69	*Megophrys maosonensis*	KIZ016045	Xiaoqiaogou Nature Reserve, Yunnan, China	KX811780	KX812080
70	*Megophrys mangshanensis*	KIZ021786	Nanling National Forest Park, Guangdong, China	KX811790	KX812079
71	*Megophrys flavipunctata*	SDBDU2009.297	East Khasi Hills dist., Meghalaya	KY022307	MH647536
72	*Megophrys glandulosa*	KIZ048439	Husa, Yunnan, China	KX811762	KX812075
73	*Megophrys medogensis*	KIZ06621	Beibeng, Xizang, China	KX811767	KX812082
74	*Megophrys periosa*	BNHS 6061	West Kameng dist., Arunachal Pradesh, IN	KY022309	MH647528
75	*Megophrys himalayana*	SDBDU2009.75	East Siang dist., Arunachal Pradesh, IN	KY022311	–
76	*Megophrys sanu*	K5198/ZSI11393	–	KX894679	–
77	*Megophrys zhangi*	KIZ014278	Zhangmu, Xizang, China	KX811765	KX812084
78	*Megophrys katabhako*	ZSIA11799	–	KX894669	–
79	*Megophrys major*	SYSa002961	Zhushihe, Yunnan, China	MH406728	MH406180
80	*Megophrys oreocrypta*	BNHS 6046	West Garo Hills dist., Meghalaya	KY022306	–
81	*Megophrys auralensis*	NCSM 79599	Aural, Kampong Speu, Cambodia	KX811807	–
82	*Megophrys parva*	SYSa003042	Zhushihe, Yunnan, China	MH406737	MH406189
83	*Megophrys dringi*	UNIMAS 8943	Gunung Mulu National Park, Sarawak, Malaysia	KJ831317	–
84	*Megophrys nankiangensis*	CIB ZYC517	Nanjiang, Sichuan, China	KX811900	–
85	*Megophrys wawuensis*	KIZ025799	Wawu Shan, Sichuan, China	KX811902	KX812062
86	*Megophrys gigantica*	SYSa003933	Wuliang shan, Yunnan, China	MH406775	MH406235
87	*Megophrys shapingensis*	KIZ014512	Liziping Nature Reserve, Sichuan, China	KX811904	KX812060
88	*Megophrys feae*	KIZ046706	Huangcaoling, Yunnan, China	KX811810	KX812056
89	*Megophrys chuannanensis*	CIB20050081	Hejiang, Sichuan, China	KM504261	–
90	*Megophrys carinense*	Tissue ID: YPX20455	Dayao Shan, Guangxi, China	KX811811	KX812057
91	*Megophrys popei*	SYS a000589	Naling Nature Reserve, Guangdong, China	KM504251	–
92	*Megophrys intermedia*	ZFMK 87596	U Bo, Phong Nha-Ke Bang NP, Vietnam	HQ588950	–
93	*Megophrys Montana*	LSUMZ 81916	Sukabumi, Java, Indonesia	KX811927	KX812163
94	*Megophrys lancip*	MZB: Amp:22233	–	KY679891	–
95	*Leptobrachium boringii*	Tissue ID: YPX37539	Emei Shan, Sichuan, China	KX811930	KX812164
96	*Leptobrachella oshanensis*	KIZ025778	Emei Shan, Sichuan, China	KX811928	KX812166

### Molecular data and phylogenetic analyses

Six adult males and one tadpole of the undescribed species, two *M. kuatunensis*, one *M. ombrophila*, and two *M. boettgeri* were included in the molecular analyses (Table 1). Total DNA was extracted using a standard phenol-chloroform extraction protocol (Sambrook et al. 1989). Two fragments of the mitochondrial 16S rRNA (16S) and cytochromeoxidase subunit I (COI) genes were amplified. For 16S, the primers P7 (5’-CGCCTGTTTACCAAAAACAT-3’) and P8 (5’-CCGGTCTGAACTCAGATCACGT-3’) were used following Simon et al. (1994), and for COI, Chmf4 (5’-TYTCWACWAAYCAYAAAGAYATCGG-3’) and Chmr4 (5’-ACYTCRGGRTGRCCRAARAATCA-3’) were used following Che et al. (2012). Gene fragments were amplified under the following conditions: an initial denaturing step at 95 °C for 4 min; 36 cycles of denaturing at 95 °C for 30 s, annealing at 52 °C (for 16S)/47 °C (for COI) for 40 s and extending at 72 °C for 70 s. Sequencing was conducted using an ABI3730 automated DNA sequencer in Shanghai DNA BioTechnologies Co., Ltd. (Shanghai, China). New sequences were deposited in GenBank (for GenBank accession numbers see Table 1).

For molecular analyses, the available sequences for congeners of *Megophrys* were downloaded from GenBank (Table 1), primarily from previous studies (Chen et al. 2017; Liu et al. 2018). For phylogenetic analyses, corresponding sequences of one *Leptobrachella oshanensis* (Liu, 1950) and one *Leptobrachium boringii* (Liu, 1945) were also downloaded (Table 1), and used as outgroups following Mahony et al. (2017). Sequences were assembled and aligned using the Clustalw module in BioEdit v.7.0.9.0 (Hall 1999) with default settings. Alignments were checked by eye and revised manually if necessary. For phylogenetic analyses of mitochondrial DNA, the dataset concatenated with 16S and COI gene sequences. To avoid under- or over-parameterization (Lemmon and Moriarty 2004; McGuire et al. 2007), the best partition scheme and the best evolutionary model for each partition were chosen for the phylogenetic analyses using PARTITIONFINDER v. 1.1.1 (Robert et al. 2012). In this analysis, 16S gene and each codon position of COI gene were defined, and Bayesian Inference Criteria was used. As a result, the analysis suggested that the best partition scheme is 16S gene/each codon position of COI gene, and selected GTR + G + I model as the best model for each partition. Phylogenetic analyses were conducted using maximum likelihood (ML) and Bayesian Inference (BI) methods, implemented in PhyML v. 3.0 (Guindon et al. 2010) and MrBayes v. 3.12 (Ronquist and Huelsenbeck 2003), respectively. For the ML tree, branch supports were drawn from 10,000 nonparametric bootstrap replicates. In BI, two runs each with four Markov chains were simultaneously run for 50 million generations with sampling every 1,000 generations. The first 25% trees were removed as the “burn-in” stage followed by calculations of Bayesian posterior probabilities (BPP) and the 50% majority-rule consensus of the post burn-in trees sampled at stationarity. Finally, mean genetic distance between *Megophrys* species based on uncorrected *p*-distance model was estimated respectively on 16S and COI genes using MEGA v. 6.06 (Tamura et al. 2013).

### Morphological comparisons

Six adult males and one tadpole of the undescribed species were measured (Table 1 and Suppl. material 1). For comparisons, six adult male specimens of *M. kuatunensis* were also measured (Supp. material 1). The terminology and methodology followed Fei et al. (2009). Measurements were taken with a dial caliper to 0.1 mm. Twenty-two morphometric characters of adult specimens were measured:

**ED** eye diameter (distance from the anterior corner to the posterior corner of the eye);

**FIL** first finger length (distance from base to tip of finger I);

**FIIL** second finger length (distance from base to tip of finger II);

**FIIIL** third finger length (distance from base to tip of finger III);

**FIVL** fourth finger length (distance from base to tip of finger IV);

**FL** foot length (distance from tarsus to the tip of fourth toe);

**HDL** head length (distance from the tip of the snout to the articulation of jaw);

**HDW** maximum head width (greatest width between the left and right articulations of jaw);

**HAL** hand length (distance from tip of third digit to proximal edge of inner palmar tubercle);

**IND** internasal distance (minimum distance between the inner margins of the external nares);

**IOD** interorbital distance (minimum distance between the inner edges of the upper eyelids);

**LAL** length of lower arm and hand (distance from the elbow to the distal end of the Finger IV);

**LW** lower arm width (maximum width of the lower arm);

**SNT** distance between the nasal the posterior edge of the vent;

**SVL** snout-vent length (distance from the tip of the snout to the posterior edge of the vent);

**SL** snout length (distance from the tip of the snout to the anterior corner of the eye);

**TFL** length of foot and tarsus (distance from the tibiotarsal articulation to the distal end of the Toe IV);

**THL** thigh length (distance from vent to knee);

**TL** tibia length (distance from knee to tarsus);

**TW** maximal tibia width;

**TYD** maximal tympanum diameter;

**UEW** upper eyelid width (greatest width of the upper eyelid margins measured perpendicular to the anterior-posterior axis).

For the single tadpole of the undescribed species, eleven morphometric characters were measured:

**BH** maximum body height;

**BW** maximum body width;

**IOS** interocular distance (minimum distance between eye);

**MW** mouth width (distance between two corners of mouth);

**SL** snout length (distance from the tip of the snout to the anterior corner of the eye);

**SS** snout to spiraculum (distance from spiraculum to the tip of the snout);

**SVL** snout-vent length;

**TAH** tail height (maximum height between upper and lower edges of tail);

**TAL** tail length (distance from base of vent to the tip of tail);

**TBW** maximum width of tail base;

**TOL** total length (distance from the tip of the snout to the tip of tail).

To reduce the impact of allometry, the correct value from the ratio of each character to SVL was calculated, and then was log-transformed for the following morphometric analyses. Mann-Whitney *U* tests were conducted to test the significance of differences on morphometric characters between the undescribed species and *M. kuatunensis*. The significance level was set at 0.05. Furthermore, principal component analyses (PCA) were conducted to highlight whether the different species were separated in morphometric space.

The new species was also compared with all other *Megophrys* species on morphology. Comparative data were obtained for related species as described in literature (Table 2).

**Table 2.** References for morphological characters for congeners of the genus *Megophrys*.

Species	Literature obtained
*M. aceras* Boulenger, 1903	Boulenger 1903
*M. acuta* Wang, Li & Jin, 2014	Li et al. 2014
*M. ancrae* Mahony, Teeling & Biju, 2013	Mahony et al. 2013
*M. angka* Wu, Suwannapoom, Poyarkov, Chen, Pawangkhanant, Xu, Jin, Murphy & Che, 2019	Wu et al. 2019
*M. auralensis* Ohler, Swan & Daltry, 2002	Ohler et al. 2002
*M. awuh* Mahony, Kamei, Teeling, & Biju, 2020	Mahony et al. 2020
*M. baluensis* (Boulenger, 1899)	Boulenger 1899a
*M. baolongensis* Ye, Fei & Xie, 2007	Ye et al. 2007
*M. binchuanensis* Ye & Fei, 1995	Ye and Fei 1995
*M. binlingensis* Jiang, Fei & Ye, 2009	Fei et al. 2009
*M. boettgeri*i (Boulenger, 1899)	Boulenger 1899b
*M. brachykolos* Inger & Romer, 1961	Inger and Romer 1961
*M. carinense* (Boulenger, 1889)	Boulenger 1889
*M. caobangensis* Nguyen, Pham, Nguyen, Luong, & Ziegler, 2020	Nguyen et al. 2020
*M. caudoprocta* Shen, 1994	Shen. 1994
*M. cheni* (Wang & Liu, 2014)	Wang et al. 2014
*M. chishuiensis* Xu, Li, Liu, Wei & Wang, 2020	Xu et al. 2020
*M. chuannanensis* (Fei, Ye & Huang, 2001)	Fei et al. 2001
*M. damrei* Mahony, 2011	Mahony 2011
*M. daweimontis* Rao & Yang, 1997	Rao and Yang 1997
*M. dongguanensis* Wang & Wang, 2019	Wang et al. 2019b
*M. dringi* Inger, Stuebing & Tan, 1995	Inger et al. 1995
*M. dzukou* Mahony, Kamei, Teeling & Biju, 2020	Mahony et al. 2020
*M. edwardinae* Inger, 1989	Inger 1989
*M. elfina* Poyarkov, Duong, Orlov, Gogoleva, Vassilieva, Nguyen, Nguyen, Nguyen, Che & Mahony, 2017	Poyarkov et al. 2017
*M. fansipanensis* Tapley, Cutajar, Mahony, Nguyen, Dau, Luong, Le, Nguyen, Nguyen, Portway, Luong & Rowley, 2018	Tapley et al. 2018
*M. feae* Boulenger, 1887	Boulenger 1887
*M. feii* Yang, Wang & Wang, 2018	Yang et al. 2018
*M. flavipunctata* Mahony, Kamei, Teeling & Biju, 2018	Mahony et al. 2018
*M. gerti* (Ohler, 2003)	Ohler 2003
*M. gigantica* Liu, Hu & Yang, 1960	Liu et al. 1960
*M. glandulosa* Fei, Ye & Huang, 1990	Fei et al. 1990
*M. hansi* (Ohler, 2003)	Ohler 2003
*M. himalayana* Mahony, Kamei, Teeling & Biju, 2018	Mahony et al. 2018
*M. hoanglienensis* Tapley, Cutajar, Mahony, Nguyen, Dau, Luong, Le, Nguyen, Nguyen, Portway, Luong & Rowley, 2018	Tapley et al. 2018
*M. huangshanensis* Fei & Ye, 2005	Fei and Ye 2005
*M. insularis* (Wang, Liu, Lyu, Zeng & Wang, 2017)	Wang et al. 2017a
*M. intermedia* Smith, 1921	Smith 1921
*M. jiangi* Liu, Li, Wei, Xu, Cheng, Wang & Wu, 2020	Liu et al. 2020
*M. jingdongensis* Fei & Ye, 1983	Fei et al. 1983
*M. jinggangensis* (Wang, 2012)	Wang et al. 2012
*M. jiulianensis* Wang, Zeng, Lyu & Wang, 2019	Wang et al. 2019b
*M. kalimantanensis* Munir, Hamidy, Matsui, Iskandar, Sidik & Shimada, 2019	Munir et al. 2019
*M. kobayashii* Malkmus & Matsui, 1997	Malkmus and Matsui 1997
*M. koui* Mahony, Foley, Biju & Teeling, 2017	Mahony et al. 2017
*M. kuatunensis* Pope, 1929	Pope 1929
*M. lancip* Munir, Hamidy, Farajallah & Smith, 2018	Munir et al. 2018
*M. leishanensis* Li, Xu, Liu, Jiang, Wei & Wang, 2018	Li et al. 2018
*M. lekaguli* Stuart, Chuaynkern, Chan-ard & Inger, 2006	Stuart et al. 2006
*M. liboensis* (Zhang, Li, Xiao, Li, Pan, Wang, Zhang & Zhou, 2017)	Zhang et al. 2017
*M. ligayae* Taylor, 1920	Taylor 1920
*M. lini* (Wang & Yang, 2014)	Wang et al. 2014
*M. lishuiensis* (Wang, Liu & Jiang, 2017)	Wang et al. 2017b
*M. longipes* Boulenger, 1886	Boulenger 1886
*M. major* Boulenger, 1908	Boulenger 1908
*M. mangshanensis* Fei & Ye, 1990	Fei et al. 2012
*M. maosonensis* Bourret, 1937	Bourret 1937
*M. medogensis* Fei, Ye & Huang, 1983	Fei et al. 1983
*M. megacephala* Mahony, Sengupta, Kamei & Biju, 2011	Mahony et al. 2011
*M. microstoma* (Boulenger, 1903)	Boulenger 1903
*M. minor* Stejneger, 1926	Stejneger 1926
*M. mirabilis* Lyu, Wang & Zhao	Lyu et al. 2020
*M. montana* Kuhl & Van Hasselt, 1822	Kuhl and Van Hasselt 1822
*M. monticola* (Günther, 1864)	Günther 1864; Mahony et al. 2018
*M. mufumontana* Wang, Lyu & Wang, 2019	Wang et al. 2019b
*M. nankiangensis* Liu & Hu, 1966	Hu and Liu 1966
*M. nankunensis* Wang, Zeng &. Wang, 2019	Wang et al. 2019b
*M. nanlingensis* Lyu, Wang, Liu & Wang, 2019	Wang et al. 2019b
*M. nasuta* (Schlegel, 1858)	Schlegel 1858
*M. numhbumaeng* Mahony, Kamei, Teeling, & Biju, 2020	Mahony et al. 2020
*M. obesa* Wang, Li & Zhao, 2014	Wang et al. 2014
*M. ombrophila* Messenger & Dahn, 2019	Messenger et al. 2019
*M. omeimontis* Liu, 1950	Liu 1950
*M. oreocrypta* Mahony, Kamei, Teeling & Biju, 2018	Mahony et al. 2018
*M. oropedion* Mahony, Teeling & Biju, 2013	Mahony et al. 2013
*M. orientalis* Li, Lyu, Wang & Wang, 2020	Li et al. 2020
*M. pachyproctus* Huang, 1981	Huang and Fei 1981
*M. palpebralespinosa* Bourret, 1937	Bourret 1937
*M. parallela* Inger & Iskandar, 2005	Inger and Iskandar 2005
*M. parva* (Boulenger, 1893)	Boulenger 1893
*M. periosa* Mahony, Kamei, Teeling & Biju, 2018	Mahony et al. 2018
*M. popei* (Zhao, Yang, Chen, Chen & Wang, 2014)	Zhao et al. 2014
*M. robusta* Boulenger, 1908	Boulenger 1908
*M. rubrimera* Tapley, Cutajar, Mahony, Chung, Dau, Nguyen, Luong & Rowley, 2017	Tapley et al. 2017
*M. sangzhiensis* Jiang, Ye & Fei, 2008	Jiang et al. 2008
*M. serchhipii* (Mathew & Sen, 2007)	Mathew and Sen 2007
*M. shapingensis* Liu, 1950	Liu 1950
*M. shimentaina* Lyu, Liu & Wang	Lyu et al. 2020
*M. shuichengensis* Tian & Sun, 1995	Tian and Sun 1995
*M. shunhuangensis* Wang, Deng, Liu, Wu & Liu, 2019	Wang et al. 2019a
*M. spinata* Liu & Hu, 1973	Hu et al. 1973
*M. stejnegeri* Taylor, 1920	Taylor 1920
*M. synoria* (Stuart, Sok & Neang, 2006)	Stuart et al. 2006
*M. takensis* Mahony, 2011	Mahony 2011
*M. tuberogranulata* Shen, Mo & Li, 2010	Mo et al. 2012
*M. vegrandis* Mahony, Teeling, Biju, 2013	Mahony et al. 2013
*M. wawuensis* Fei, Jiang & Zheng, 2001	Fei et al. 2012
*M. wugongensis* Wang, Lyu & Wang, 2019	Wang et al. 2019b
*M. wuliangshanensis* Ye & Fei, 1995	Ye and Fei 1995
*M. wushanensis* Ye & Fei, 1995	Ye and Fei 1995
*M. xianjuensis* Wang, Wu, Peng, Shi, Lu & Wu, 2020	Wang et al. 2020
*M. xiangnanensis* Lyu, Zeng & Wang	Lyu et al. 2020
*M. yangmingensis* Lyu, Zeng & Wang	Lyu et al. 2020
*M. zhangi* Ye & Fei, 1992	Ye and Fei 1992
*M. zunhebotoensis* (Mathew & Sen, 2007)	Mathew and Sen 2007

### Bioacoustics analyses

The advertisement calls of the undescribed species were recorded from the holotype specimen CIBQY20200726001 in the field on 26 July 2020 from Qingyuan County, Zhejiang Province, China. When registrating the male in the stream the ambient air temperature was 21.5 °C and there was air humidity of 87%. For comparisons, the advertisement calls of *M. kuatunensis* from Wuyi Mountain, Fujian Province, China were recorded from the specimens CIBWY18082410, CIBWY18082411 and CIBWY18082412 at an ambient air temperature of 22.0 °C and air humidity of 88% on 24 August 2018. SONY PCM-D50 digital sound recorder was used to record within 20 cm of the calling individual. The sound files in wave format were resampled at 48 kHz with sampling depth 24 bits. The sonograms and waveforms were generated by WaveSurfer software (Sjöander and Beskow 2000) from which all parameters and characters were measured. Ambient temperature was taken by a digital hygrothermograph.

## Results

### Phylogenetic analyses

**Figure 2.**
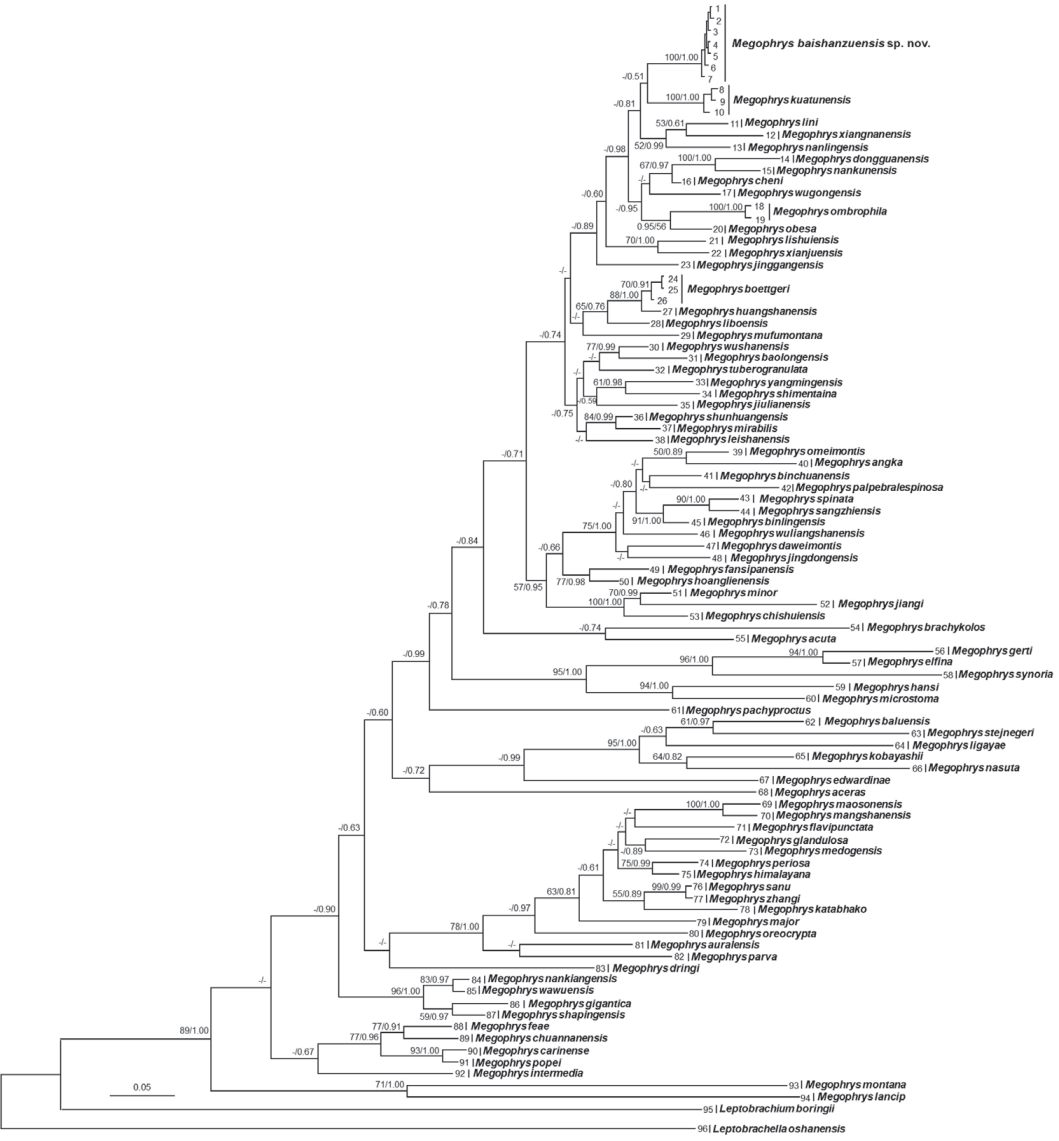
Maximum likelihood (ML) tree of the genus *Megophrys* reconstructed based on 16S rRNA and COI gene sequences. Bayesian posterior probability/ML bootstrap supports were denoted beside each node. Samples 1–96 refer to Table 1.

Aligned sequence matrix of 16S+COI contains 1104 bp. ML and BI trees of the mitochondrial DNA dataset presented almost consistent topology, and as well, though relationships of many clades were unresolved (Fig. 2). In mitochondrial DNA trees, all samples of the undescribed species were clustered into one clade which was deeply clustered into the *Megophrys* clade. The species is likely sister to *M. kuatunensis* (bootstrap supports < 50% and BPP = 0.51) though the relationships between the two species and most other congeners were not resolved (all bootstrap supports < 50% and many BPP < 0.95).

Genetic distances based on 16S and COI genes with uncorrected *p*-distance model between the samples of the undescribed species were all below 0.2%. The genetic distance between the undescribed species and its closest related species *M. kuatunensis* were 2.1% and 8.1% on 16S and COI respectively, which was higher or at the same level with those among many pairs of sister species, for example, 1.7% and 3.8% on 16S and COI respectively between *M. spinata* and *M. sangzhiensis* (Suppl. materials 2 and 3).

**Figure 3.**
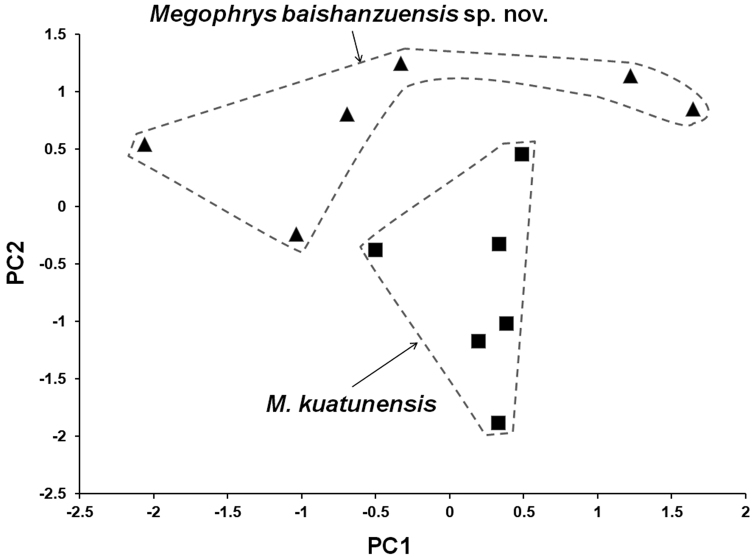
Plots of the first principal component (PC1) versus the second (PC2) for *Megophrys baishanzuensis* sp. nov. and *M. kuatunensis* from principal component analyses on male group.

In PCA for male group, the total variation of the first two principal components was 47.5%. On the two-dimensional plots of PC1 vs. PC2, the undescribed species was almost separated from *M. kuatunensis* (Fig. 3). The first two principal component axes could separate *M. kuatunensis* from the undescribed species mainly based on limb and head characteristics, namely, HDL, HDW, IND, FIL, FIIL and FL. The results of Mann-Whitney *U* tests indicated that in males, the undescribed species was significantly different from *M. kuatunensis* on UEW and TFL (*p*-values < 0.05; Table 3).

**Table 3.** Morphometric comparisons between the adult specimens of *Megophrys baishanzuensis* sp. nov. and *M. kuatunensis*. Units given in mm. See abbreviations for the morphological characters in Materials and methods section. P-value resulted from Mann-Whitney *U* test. Significant level at 0.05.

Character	*Megophrys baishanzuensis* sp. nov.		*M. kuatunensis*		Mann-Whitney U value	P-value
	Male (N = 6)		Male (N = 6)			
	Ranging	Mean ± SD	Ranging	Mean ± SD		
SVL	28.4–32.4	30.5 ± 1.8	28.4–32.4	30.5 ± 1.8	13.000	0.423
HDL	8.0–9.1	8.6 ± 0.4	8.0–9.1	8.6 ± 0.4	6.000	0.055
HDW	9.3–10.5	10.2 ± 0.4	9.3–10.5	10.2 ± 0.4	8.000	0.109
SL	3.4–4.1	3.8 ± 0.3	3.4–4.1	3.8 ± 0.3	16.000	0.749
SNT	1.5–2.6	2.0 ± 0.4	1.5–2.6	2.0 ± 0.4	18.000	1.000
IND	3.1–3.7	3.4 ± 0.3	3.1–3.7	3.48 ± 0.3	16.000	0.749
IOD	2.8–3.3	3.0 ± 0.2	2.8–3.3	3.08 ± 0.2	6.000	0.055
UEW	2.3–3.0	2.6 ± 0.2	2.3–3.0	2.6 ± 0.2	2.000	**0.010**
ED	3.7–4.0	3.8 ± 0.1	3.7–4.0	3.8 ± 0.1	15.000	0.631
TYD	1.5–2.1	1.8 ± 0.2	1.5–2.1	1.8 ± 0.2	16.000	0.749
LAL	13.4–14.6	14.1 ± 0.5	13.4–14.6	14.2 ± 0.5	9.000	0.150
HAL	6.6–7.9	7.1 ± 0.5	6.6–7.9	7.1 ± 0.5	6.000	0.055
LW	2.2–2.7	2.4 ± 0.2	2.2–2.7	2.4 ± 0.2	10.000	0.200
FIL	2.2–2.8	2.5 ± 0.2	2.2–2.8	2.5 ± 0.2	17.000	0.873
FIIL	2.4–3.0	2.7 ± 0.2	2.4–3.0	2.7 ± 0.2	12.000	0.200
FIIIL	4.3–5.1	4.6 ± 0.3	4.3–5.1	4.6 ± 0.3	10.000	0.200
FIVL	2.6–3.6	3.0 ± 0.4	2.6–3.6	3.0 ± 0.4	15.000	0.631
THL	12.2–13.5	12.9 ± 0.5	12.2–13.5	12.9 ± 0.5	10.000	0.200
TL	12.8–14.9	13.9 ± 0.9	12.8–14.9	13.9 ± 0.9	13.000	0.423
TW	2.7–4.2	3.3 ± 0.5	2.7–4.2	3.3 ± 0.5	13.000	0.423
TFL	17.8–20.4	19.4 ± 1.0	17.8–20.4	19.4 ± 1.0	1.000	**0.006**
FL	11.2–12.3	11.8 ± 0.4	11.2–12.3	11.8 ± 0.4	13.000	0.423

**Figure 4.**
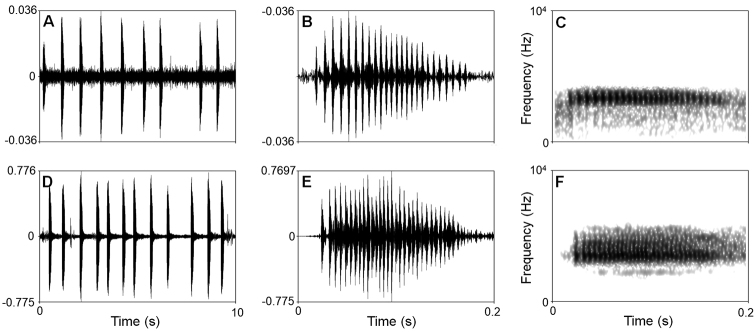
Visualization of advertisement calls of *Megophrys baishanzuensis* sp. nov. and *M. kuatunensis***A–C** waveform showing 10 seconds, waveform showing 0.2 seconds and sonogram showing 0.2 seconds of CIBQY20200726001 of *Megophrys baishanzuensis* sp. nov. **D–F** waveform showing 10 seconds, waveform showing 0.2 seconds and sonogram showing 0.2seconds of CIBWY18082410 of *M. kuatunensis*.

There were two differences in sonograms and waveforms of calls between the undescribed species and *M. kuatunensis* (Fig. 4; Table 4). Firstly, the undescribed species had slower call repetition rate than the latter (0.79 call/s in the former vs. 1.18 call/s in the latter). Secondly, the undescribed species had lower dominant frequency (3.19–3.38 kHz in the former vs. 3.38–3.75 kHz in the latter).

**Table 4.** Comparisons of characteristics of advertisement calls of *Megophrys baishanzuensis* sp. nov. and *M. kuatunensis*.

**Call character**	***Megophrys baishanzuensis* sp. nov.**	***M. kuatunensis***		
	CIBQY20200726001	CIBWY2018082410	CIBWY2018082412	WY2018082411
**Number of call groups measured**	11	30	30	20
**Number of notes measured**	22	30	30	40
**Call duration (ms)**	151.0–170.0 (162.4 ± 5.7)	131.0–163.0 (147.2 ± 7.1)	131.0–163.0 (147.2 ± 7.1)	130.0–159.0 (120.9 ± 5.9)
**Call repetition rate (calls/s)**	0.79	1.18	1.13	1.3
**Intercall interval (ms)**	682.0–1869.0 (936.8 ±349.0)	404–1548.0 (687.3 ± 206.8)	404–1548.0 (687.3 ± 206.8)	350.0–733.0 (458.4 ± 87.1)
**Pulses/call**	23.0–30.0 (26.0 ± 2.4)	25.0–36.0 (30.0 ± 2.3)	25.0–36.0 (30.0 ± 2.3)	32.0–40.4 (35.7 ± 2.3)
**Dominant frequency (kHz)**	3.19–3.38 (3.36 ± 0.06)	3.38–3.75 (3.46 ± 0.16)	3.38–3.75 (3.46 ± 0.16)	3.38–3.38 (3.38±0.01)
**Pulse duration (ms)**	3.0–6.0 (4.9 ± 0.6)	3.0–6.0 (4.4 ± 0.7)	3.0–6.0 (4.4 ± 0.7)	3.0–6.0 (4.5 ± 0.6)

Based on the molecular phylogenetic analyses, morphological comparisons (Supp. material 4), and bioacoustics differences, the specimens from Qiangyuan County, Zhejiang Province, China represent a new species which is described as follows.

### Taxonomic accounts

#### Megophrys baishanzuensis sp. nov.

Taxon classificationAnimaliaAnuraMegophryidae

C4F90C1D-CF76-5BBD-9E7B-75E7B8040B2C

http://zoobank.org/563EBE4E-45FF-4956-AB3B-70467B2D338E

**Figure 5.**
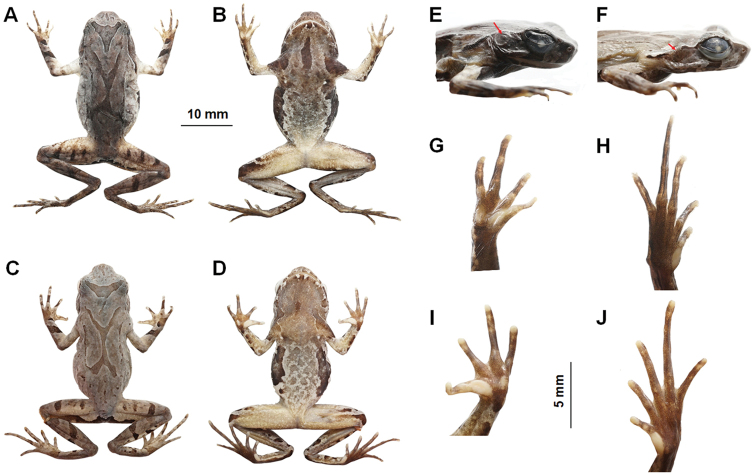
Photos of the holotype specimen CIBQY20200726001 of *Megophrys baishanzuensis* sp. nov. and topotype specimen of *M. kuatunensis***A, B, E, G, H** dorsal view of body, ventral view of body, lateral view of head, ventral view of hand, and ventral view of foot of CIBQY20200726001, respectively **C, D, F, I, J** dorsal view of body, ventral view of body, lateral view of head, ventral view of hand, and ventral view of foot of CIBWY18082413, respectively. Red arrow points to tympanum.

**Figure 6.**
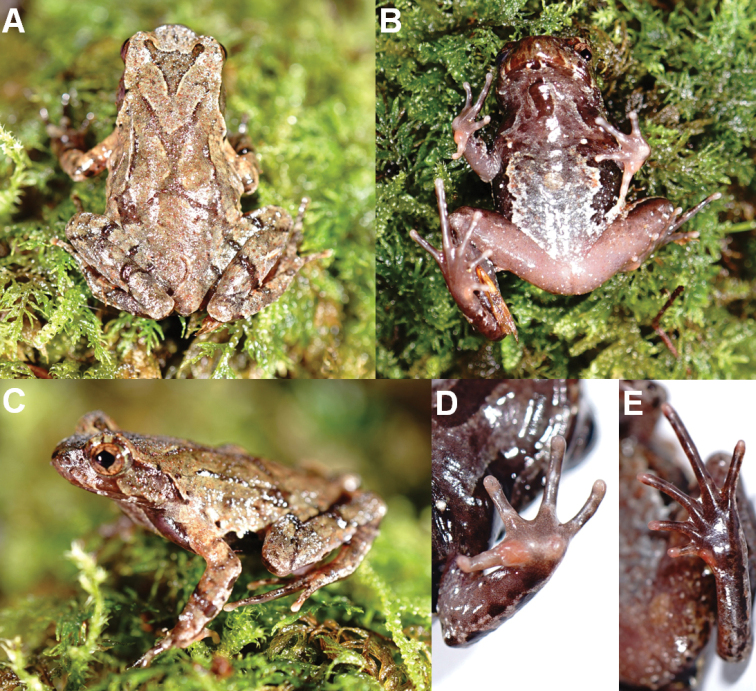
Photos of the holotype CIBQY20200726001 of *Megophrys baishanzuensis* sp. nov. in life **A** dorsal view **B** ventral view **C** lateral view **D** ventral view of hand **E** ventral view of foot.

**Figure 7.**
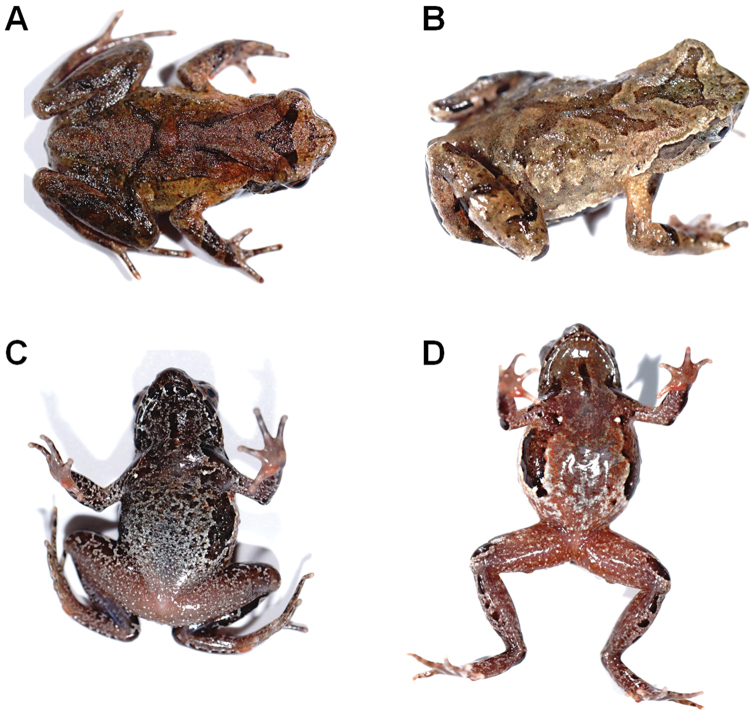
Color variation in *Megophrys baishanzuensis* sp. nov. in life **A** dorsal view of the adult male CIBQY2020200719001 **B** dorsal view of the adult male CIBQY2020200719004 **C** ventral view of the adult male CIBQY2020200719003 **D** ventral view of the adult male CIBQY2020200726002.

**Figure 8.**
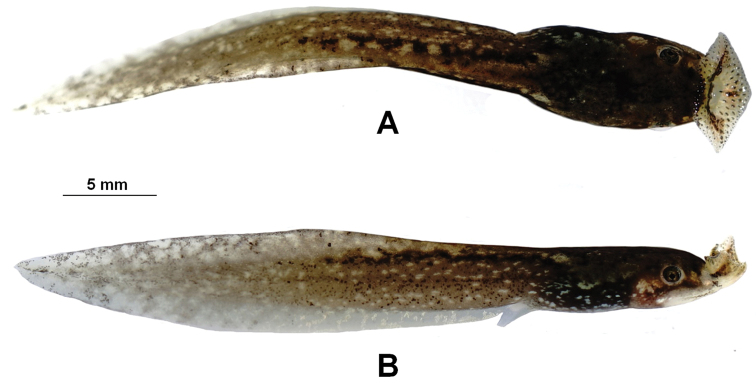
Photos of the tadpole CIBQY20200719006 of *Megophrys baishanzuensis* sp. nov. in life **A** dorsal view **B** lateral view.

Figs 4A, B, E, G, H , 5 , 6 , 7 , 8 ; Tables 1 , 2 , 3 , 4 , Suppl. materials 1 , , , 4

##### Holotype.

CIBQY20200726001 (Figs 4A, B, E, G, H, 5), adult male, from Baishanzu National Park, Qingyuan County, Zhejiang Province, China (27.76°N, 119.18°E, ca. 1537 m a.s.l.), collected by Bin Wang on 26 July 2020.

##### Paratype.

Five adult males collected from the same place as holotype collected by Bin Wang. CIBQY20200719001-CIBQY20200719004 collected on 19 July 2020 by Bin Wang, and CIBQY20200726002 collected by Zhonghao Luo on 26 July 2020.

##### Other material examined.

One tadpole (CIBQY20200719005; Fig. 7) collected by Bin Wang on 19 July 2020.

##### Diagnosis.

*Megophrys baishanzuensis* sp. nov. is assigned to the genus *Megophrys* based on molecular phylogenetic analyses and the following generic diagnostic characters: snout shield-like; projecting beyond the lower jaw; canthus rostralis distinct; chest glands small and round, closer to the axilla than to midventral line; femoral glands on rear part of thigh; vertical pupils (Fei et al. 2009).

*Megophrys baishanzuensis* sp. nov. could be distinguished from its congeners by a combination of the following morphological characters: body size small (SVL 28.4–32.4 mm in males); vomerine teeth absent; tongue not notched behind; tympanum distinctly visible, oval; a small horn-like tubercle at the edge of each upper eyelid; two metacarpal tubercles distinctly visible in hand; toes without webbing; heels overlapping when thighs are positioned at right angles to the body; tibiotarsal articulation reaching the level to the middle of eye when leg stretched forward.

##### Description of holotype.

(Figs 4A, B, E, G, H, 5). SVL 28.5 mm; head width larger than head length (HDW/HDL ratio ca. 1.3); snout obtusely pointed, protruding well beyond the margin of the lower jaw in ventral view; loreal region vertical and concave; canthus rostralis well-developed; top of head flat in dorsal view; eye large, eye diameter 46.0% of head length; pupils vertical; nostril orientated laterally, closer to snout than eye; tympanum distinct, 55.8% of eye diameter; vomerine ridges present and vomerine teeth absent; margin of tongue smooth, not notched behind.

Forelimbs slender, the length of lower arm and hand 47.0% of SVL; fingers slender, relative finger lengths: I < II < IV < III; tips of digits globular, without lateral fringes; subarticular tubercle distinct at the base of each finger; two metacarpal tubercles, prominent, oval-shaped, the inner one bigger than the outer one.

Hindlimbs slender, tibia length 46.5% times of SVL; heels overlapping when thighs are positioned at right angles to the body, tibiotarsal articulation reaching the middle of eye when leg stretched forward; tibia length longer than thigh length; relative toe lengths I < II < V < III < IV; tips of toes round, slightly dilated; subarticular tubercles absent on each toes; toes without webbing but with narrow lateral fringe; inner metatarsal tubercle oval-shaped; outer metatarsal tubercle absent.

Dorsal skin rough, several large warts scattered on flanks; a small horn-like tubercle at the edge of each upper eyelid; tubercles on the dorsum forming a X-shaped ridge, two dorsolateral parallel ridges on either side of the X-shaped ridges; an inverted triangular brown speckle between two upper eyelidsseveral tubercles scattered on dorsal, flanks and dorsal surface of thighs and tibias; supratympanic fold distinct.

Numerous granules scattered on ventrum; pectoral and femoral glands distinct; numerous white granules on outer thighs.

***Coloration of holotype in life.*** (Fig. 5). Dorsal brown, several pink tubercles scattered on dorsal, an inverted triangular brown speckle between the eyes; X-shaped ridges on the dorsum brown, four dark transverse bands on the dorsal surface of the thigh and shank; ventral surface of body white with brown spots; two dark brown dark bars on the flanks, throat brown; white vertical bars on lower and upper lip; ventral surface of anterior limb dark reddish purple, posterior limb orange with numerous white granules; tip of digits pale grey; inner metatarsal tubercle and two metacarpal tubercles pinkish; soles uniform dark reddish purple; pectoral glands white.

***Coloration of holotype in preservation.*** (Fig. 4A, B, E, G, H). Color of dorsal surface fades to taupe; the inverted triangular brown speckle between the eyes and brown X-shaped ridges on dorsum are more distinct; ventral surface greyish white; creamy-white substitutes the purple grey on tip of digits; the posterior of ventral surface of body, inner of thigh and upper of tibia fades to creamy-white.

***Variation.*** Fig. 6. Measurements and basic statistics of adult specimens are presented in Tables 3 and Supp. material 1. All specimens were similar in morphology but some individuals different from the holotype in color pattern. In CIBQY2020200719001 the tubercles on the dorsum forming two ≻ shaped, disconnected ridges (Fig. 6A); in CIBQY2020200719004 the tubercles on the dorsum forming a big and distinct X-shaped speckle (Fig. 6B); in CIBQY2020200719003 ventral surface of body grey with brown spots (Fig. 6C); in CIBQY2020200726002 ventral surface of body and limbs brownish red (Fig. 6D).

***Tadpole description.*** Fig. 7. The tadpole CIBQY20200719006 (Fig. 7) was confirmed as *Megophrys baishanzuensis* sp. nov by molecular phylogenetic analyses. Measurements in mm. Stage 31. Body slender, body brownish black and tail pale brown, body height greater than tail height; dorsal fin arising behind the origin of the tail, the highest fin near mid-length, tapering gradually to the narrowly pointed tip; tail approximately 1.9 times as long as snout-vent length; tail height 13.6% of tail length; body width longer than body height (BW/BH1.2); eyes large, lateral, nostril near eyes; spiracle on the left side of the body and distinct; oral disk terminal, lips expanded and directed upwardly into a umbelliform oral disk; flank of body brownish black with some white spots, tail fins lightly colored, with small white and black spots. TOL 22.7; SVL 8.7; BW 3.0; BH 2.7; SL 2.0; SS 4.0; IOS 1.8; TAL 14.7; TAH 2.2; TBD 1.5; MW 1.3.

***Advertisement call.*** Fig. 4. The call description is based on recordings of the holotype CIBQY20200726001 (Fig. 4; Table 4) from a shrub leaf near the streamlet. Call duration was 151.0–170.0 ms (mean 162.4 ± 5.7). Inter-call interval was 682.0–1869.0 ms (mean 936.8 ± 349.0). Pulse/call was 23.0–30.0 (mean 26.0 ± 2.4); pulse duration was 3.0–6.0 (mean 4.9 ± 6.0) and call repetition rate was 0.79 call/s.

Amplitude modulation within note was apparent, beginning with moderately high energy pulses, increasing to the maximum by approximately quarter, and then decreasing towards the end. The average dominant frequency was 3.36 ± 0.06 (3.19–3.38 kHz).

***Secondary sexual characters.*** A single subgular vocal sac present in male. In breeding season, nuptial pads are present on the dorsal base of the first two fingers in males.

##### Comparisons.

Supp. material 4. By having small body size, *Megophrys baishanzuensis* sp. nov. differs from *M. ancrae*, *M. auralensis*, *M. awuh*, *M. baluensis*, *M. baolongensis*, *M. binlingensis*, *M. boettgeri*, *M. caobangensis*, *M. carinense*, *M. caudoprocta*, *M. chishuiensis*, *M. chuannanensis*, *M. damrei*, *M. daweimontis*, *M. dzukou*, *M. edwardinae*, *M. feae*, *M. flavipunctata*, *M. gigantica*, *M. glandulosa*, *M. hansi*, *M. himalayana*, *M. hoanglienensis*, *M. huangshanensis*, *M. insularis*, *M. jiangi*, *M. jingdongensis*, *M. jinggangensis*, *M. kalimantanensis*, *M. kobayashii*, *M. lancip*, *M. lekaguli*, *M. liboensis*, *M. ligayae*, *M. lini*, *M. longipes*, *M. major*, *M. mangshanensis*, *M. medogensis*, *M. megacephala*, *M. mirabilis*, *M. montana*, *M. monticola*, *M. nasuta*, *M. obesa*, *M. omeimontis*, *M. orientalis*, *M. pachyproctus*, *M. palpebralespinosa*, *M. parallela*, *M. parva*, *M. periosa*, *M. platyparietus*, *M. popei*, *M. sangzhiensis*, *M. serchhipii*, *M. shapingensis*, *M. shuichengensis*, *M. spinata*, *M. takensisM. wawuensis*, and *M. xiangnanensis* (maximum SVL < 33.0 mm in the new species vs. minimum SVL > 34.0 mm in the latter).

By vomerine teeth absent, *Megophrys baishanzuensis* sp. nov. differs from *M. ancrae*, *M. baluensis*, *M. carinense*, *M. caudoprocta*, *M. chuannanensis*, *M. damrei*, *M. daweimontis*, *M. dongguanensis*, *M. dzukou*, *M. fansipanensis*, *M. feae*, *M. flavipunctata*, *M. glandulosa*, *M. himalayana*, *M. hoanglienensis*, *M. insularis*, *M. intermedia*, *M. jingdongensis*, *M. jinggangensis*, *M. jiulianensis*, *M. kalimantanensis*, *M. kobayashii*, *M. lancip*, *M. lekaguli*, *M. liboensis*, *M. ligayae*, *M. longipes*, *M. mangshanensis*, *M. maosonensis*, *M. medogensis*, *M. megacephala*, *M. montana*, *M. nankunensis*, *M. nanlingensis*, *M. nasuta*, *M. numhbumaeng*, *M. omeimontis*, *M. oreocrypta*, *M. orientalis*, *M. oropedion*, *M. pachyproctus*, *M. palpebralespinosa*, *M. parallela*, *M. parva*, *M. periosa*, *M. platyparietus*, *M. popei*, *M. robusta*, *M. rubrimera*, *M. serchhipii*, M. *shimentaina*, *M. stejnegeri*, *M. takensis*, *M. zhangi*, and *M. zunhebotoensis* (vs. present in the latter).

By a small horn-like tubercle present at the edge of each upper eyelid, *Megophrys baishanzuensis* sp. nov. differs from *M. aceras*, *M. acuta*, *M. carinense*, *M. caudoprocta*, *M. chuannanensis*, *M. feae*, *M. gerti*, *M. hansi*, *M. intermedia*, *M. intermedia*, *M. jinggangensis*, *M. kalimantanensis*, *M. koui*, *M. lancip*, *M. liboensis*, *M. microstoma*, *M. montana*, *M. nasuta*, *M. orientalis*, *M. palpebralespinosa*, *M. platyparietus*, *M. popei*, *M. shuichengensis*, *M. stejnegeri*, and *M. synoria* (vs. having a prominent and elongated tubercle in the latter).

By tongue not notched behind, *Megophrys baishanzuensis* sp. nov. differs from *M. ancrae*, *M. baolongensis*, *M. binlingensis*, *M. boettgeri*, *M. carinense*, *M. cheni*, *M. chuannanensis*, *M. damrei*, *M. dringi*, *M. dzukou*, *M. fansipanensis*,*M. feae*, *M. feii*, *M. flavipunctata*, *M. gerti*, *M. glandulosa*, *M. hoanglienensis*, *M. huangshanensis*, *M. insularis*, *M. jiulianensis*. *M. jingdongensis*, *M. kalimantanensis*, *M. kuatunensis*, *M. liboensis*, *M. mangshanensis*, *M. maosonensis*, *M. medogensis*, *M. minor*, *M. nankiangensis*, *M. nanlingensis*, *M. numhbumaeng*, *M. omeimontis*, *M. oropedion*, *M. pachyproctus*, *M. parallela*, *M. popei*, *M. robusta*, *M. sangzhiensis*, *M. shapingensis*, *M. shuichengensis*, *M. spinata*, *M. vegrandis*, *M. wawuensis*, *M. zhangi*, and *M. zunhebotoensis* (vs. notched behind in the latter).

By toes with narrow lateral fringes, *Megophrys baishanzuensis* sp. nov. differs from *M. angka*, *M. baolongensis*, M. brachykolos, *M. caobangensis*, *M. chishuiensis*, *M. damrei*, *M. daweimontis*, *M. dongguanensis*, *M. fansipanensis*, *M. feae*, *M. himalayana*, *M. hoanglienensis*, *M. huangshanensis*, *M. insularis*, *M. jiangi*, *M. jiulianensis*, *M. kalimantanensis*, *M. koui*, *M. leishanensis*, *M. lekaguli*, *M. lishuiensis*, *M. major*, *M. mangshanensis*, *M. medogensis*, *M. megacephala*, *M. microstoma*, *M. minor*, *M. nankunensis*, *M. obesa*, *M. ombrophila*, *M. oreocrypta*, *M. oropedion*, *M. pachyproctus*, *M. parva*, *M. periosa*, *M. shunhuangensis*, *M. takensis*, *M. tuberogranulata*, *M. wawuensis*, *M. wugongensis*, *M. wuliangshanensis* and *M. xianjuensis* (vs. lacking in the latter); and differs from *M. binchuanensis*, *M. boettgeri*, *M. carinense*, *M. cheni*, *M. chuannanensis*, *M. dringi*, *M. feii*, *M. gigantica*, *M. glandulosa*, *M. intermedia*, *M. jingdongensis*, *M. liboensis*, *M. lini*, *M. orientalis*, *M. palpebralespinosa*, *M. platyparietus*, *M. shapingensis*, *M. shuichengensis*, M. *spinata*, and *M. xiangnanensis* (vs. with wide lateral fringes in the latter).

By toes without webbing, *Megophrys baishanzuensis* sp. nov. differs from *M. brachykolos*, *M. carinense*, *M. flavipunctata*, *M. jingdongensis*, *M. jinggangensis*, *M. lini*, *M. major*, *M. palpebralespinosa*, *M. popei*, *M. shuichengensis*, and *M. spinata* (vs. at least one-fourth webbed in the latter).

By heels overlapping when thighs are positioned at right angles to the body, *Megophrys baishanzuensis* sp. nov. differs from *M. actuta*, *M. brachykolos*, *M. dongguanensis*, *M. huangshanensis*, *M. kuatunensis*, *M. nankunensis*, *M. obesa*, *M. ombrophila*, *M. wushanensis*, and *M. wugongensis* (vs. just meeting or not meeting in the latter).

By tibiotarsal articulation reaching to the level to the middle of eye when leg stretched forward, *Megophrys baishanzuensis* sp. nov. differs from *M. daweimontis*, *M. glandulosa*, *M. lini*, *M. major*, *M. medogensis*, *M. obesa*, *M. sangzhiensis*, and *M. yangmingensis* (vs. reaching the anterior corner of the eye or beyond eye or nostril and tip of snout in the latter); differs from *M. mufumontana* (vs. reaching tympanum in males and to the eye in females in the latter); and differs from *M. chishuiensis* (vs. reaching the level between tympanum and eye in the latter).

By having an internal single subgular vocal sac in male, *Megophrys baishanzuensis* sp. nov. differs from *M. caudoprocta*, *M. shapingensis*, and *M. shuichengensis* (vs. vocal sac absent in the latter).

The congeners *M. boettgeri*, *M. lishuiensis*, *M. ombrophila*, and *M. xianjuensis* all occur in Wuyi Mountains, Fujian Province and/or Zhejiang Province, China, and probably have sympatric distribution with *Megophrys baishanzuensis* sp. nov. (Fei et al. 2012; Wang et al. 2017b; Messenger et al. 2019; Wang et al. 2020). The new species can be distinguished from these species by a series of morphological characters as follows. The new species differs from *M. boettgeri* by body size smaller (adult males with 28.4–32.4 mm vs. adult males with 34.5–37.8 mm), and in breeding male nuptial pads present on the dorsal base of the first two fingers (vs. nuptial pad only on the first finger). The new species differs from *M. lishuiensis* by vomerine ridges present (vs. absent), toes with narrow lateral fringe (vs. without), and tibiotarsal articulation reaching the middle of eye when leg stretched forward (vs. reaching the range from tympanum to eye). The new species differs from *M. ombrophila* by heels overlapping when thighs are positioned at right angles to the body (vs. not meeting), vomerine ridges present (vs. absent), and toes with narrow lateral fringe (vs. without). The new species differs from *M. xianjuensis* by tibiotarsal articulation reaching the middle of eye when leg stretched forward (vs. reaching the range from tympanum to eye), and toes with narrow lateral fringe (vs. without).

*Megophrys baishanzuensis* sp. nov. is phylogenetically closest to *M. kuatunensis*. *Megophrys baishanzuensis* sp. nov. could be identified from *M. kuatunensis* distinctly by tibiotarsal articulation reaching the middle of eye when leg stretched forward (vs. reaching the range from tympanum to eye), heels overlapping when thighs are positioned at right angles to the body (vs. not meeting), tongue not notched behind (vs. notched feebly), the supratympanic fold more expanded in dorsal view and tympanum protruding (vs. concave), and having significantly lower ratios of UEW and TFL to SVL in males (all *p*-values < 0.05; Table 3). On call characters, the new species has slower call repetition rate (0.79 call/s in the new species vs. 1.18 call/s in *M. kuatunensis*), and has lower dominant frequency (3.19–3.38 kHz in the new species vs. 3.38–3.75 kHz in *M. kuatunensis*).

##### Distribution and habitat.

**Figure 9.**
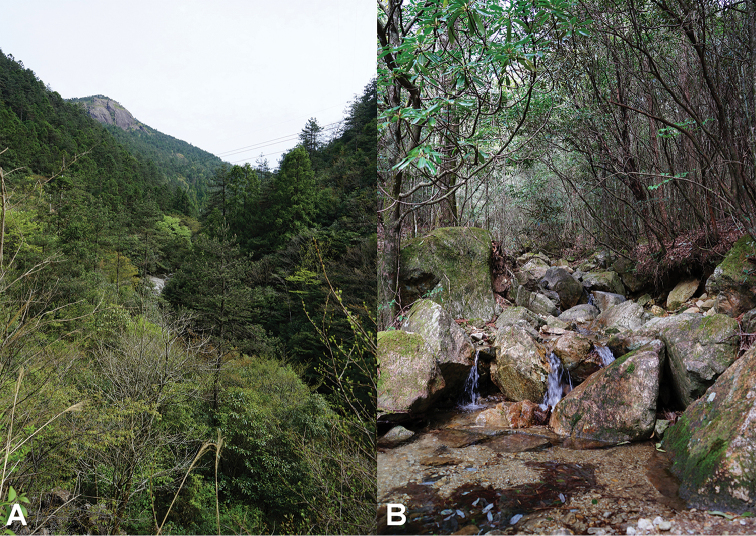
Habitats of *Megophrys baishanzuensis* sp. nov. in the type locality, Baishanzu Naitonal Park, Qingyuan County, Zhejiang Province, China **A** landscape for forest **B** the stream under the forest inhabited by *Megophrys baishanzuensis* sp. nov.

*Megophrys baishanzuensis* sp. nov. is known from the type locality, Baishanzu National Park, Qingyuan County Zhejiang Province, China, at elevations between 1400–1600 m. The individuals of the new species were frequently found in the stream surrounded by evergreen broadleaved forests (Fig. 9). *M. boettgeri* was also found in the same stream.

##### Etymology.

The specific name *baishanzuensis* refers to the distribution of this species, Baishanzu National Park, Qingyuan County, Zhejiang Province, China. We propose the common name “Baishanzu horned toad” (English) and Bai Shan Zu Jiao Chan (百山祖角蟾, Chinese).

## Discussion

Although *Megophrys baishanzuensis* sp. nov. superficially resembles *M. kuatunensis*, molecular phylogenetic analyses, detailed morphological comparisons and call datas all proposed the distinct differences between them. Moreover, the breeding seasons of them are different. According to our surveys, the breeding season of *M. kuatunensis* is in April to May in Wuyi Mountain, Fujian Province, China. But in this season, we did not find any individual of *Megophrys baishanzuensis* sp. nov. in Qingyuan County, Zhejiang Province, China. And, the breeding season of the new species should be later than June because in June, we only listened to the calls of one male in the type locality (< 10 °C), and, in late July, the males of the species started to call when the temperature was just higher than 18 °C (but we did not find any female individual and egg of it). Different call characteristics and breeding ecology most probably promoted separation of the two species.

During our several and extensive surveys, we only found fewer than 15 adult males of *Megophrys baishanzuensis* sp. nov., only in a small stream near the top of the mountain in Baishanzu National Park, Zhejiang Province, China, and even then, we did not find any female, and only found four tadpoles of this species. Obviously, the population of the new species is very endemic and small. Fortunately, this population is in a preserved area in Baishanzu National Park. Of course, we still should make a reinforced plan to preserve this area for this toad species.

## Supplementary Material

XML Treatment for Megophrys baishanzuensis

## Supplementary materials

Supplementary material 1 Table S1 Data type morphological measurements Explanation note Measurements of the adult specimens of *Megophrys baishanzuensis* sp. nov. and *M. kuatunensis*. Units given in mm. See abbreviations for the morphological characters in Materials and methods section. https://binary.pensoft.net/file/488591This dataset is made available under the Open Database License (http://opendatacommons.org/licenses/odbl/1.0/). The Open Database License (ODbL) is a license agreement intended to allow users to freely share, modify, and use this Dataset while maintaining this same freedom for others, provided that the original source and author(s) are credited.Bin Wang

Supplementary material 2 Table S2 Data type genetic distance Explanation note Uncorrected *p*-distances between the *Megophrys* species on the 16S gene. https://binary.pensoft.net/file/488592This dataset is made available under the Open Database License (http://opendatacommons.org/licenses/odbl/1.0/). The Open Database License (ODbL) is a license agreement intended to allow users to freely share, modify, and use this Dataset while maintaining this same freedom for others, provided that the original source and author(s) are credited.Bin Wang

Supplementary material 3 Table S3 Data type genetic distance Explanation note Uncorrected *p*-distances between the *Megophrys* species on the COI gene. https://binary.pensoft.net/file/488593This dataset is made available under the Open Database License (http://opendatacommons.org/licenses/odbl/1.0/). The Open Database License (ODbL) is a license agreement intended to allow users to freely share, modify, and use this Dataset while maintaining this same freedom for others, provided that the original source and author(s) are credited.Bin Wang

Supplementary material 4 Table S4 Data type morphological comparisons Explanation note Diagnostic characters separating *Megophrys baishanzuensis* sp. nov. from other species of *Megophrys*. https://binary.pensoft.net/file/488594This dataset is made available under the Open Database License (http://opendatacommons.org/licenses/odbl/1.0/). The Open Database License (ODbL) is a license agreement intended to allow users to freely share, modify, and use this Dataset while maintaining this same freedom for others, provided that the original source and author(s) are credited.Bin Wang
